# MutEnricher: a flexible toolset for somatic mutation enrichment analysis of tumor whole genomes

**DOI:** 10.1186/s12859-020-03695-z

**Published:** 2020-07-31

**Authors:** Anthony R. Soltis, Clifton L. Dalgard, Harvey B. Pollard, Matthew D. Wilkerson

**Affiliations:** 1 The American Genome Center, Collaborative Health Initiative Research Program, Uniformed Services University of the Health Sciences, Bethesda, MD USA; 2 Henry M. Jackson Foundation for the Advancement of Military Medicine, Bethesda, MD USA; 3 Department of Anatomy, Physiology, and Genetics, Uniformed Services University of the Health Sciences, Bethesda, MD USA

## Abstract

**Background:**

Analysis of somatic mutations from tumor whole exomes has fueled discovery of novel cancer driver genes. However, ~ 98% of the genome is non-coding and includes regulatory elements whose normal cellular functions can be disrupted by mutation. Whole genome sequencing (WGS), on the other hand, allows for identification of non-coding somatic variation and expanded estimation of background mutation rates, yet fewer computational tools exist for specific interrogation of this space.

**Results:**

We present MutEnricher, a flexible toolset for investigating somatic mutation enrichment in both coding and non-coding genomic regions from WGS data. MutEnricher contains two distinct modules for these purposes that provide customizable options for calculating sample- and feature-specific background mutation rates. Additionally, both MutEnricher modules calculate feature-level and local, or “hotspot,” somatic mutation enrichment statistics.

**Conclusions:**

MutEnricher is a flexible software package for investigating somatic mutation enrichment that is implemented in Python, is freely available, can be efficiently parallelized, and is highly configurable to researcher's specific needs. MutEnricher is available online at https://github.com/asoltis/MutEnricher.

## Background

Analysis of somatic mutations throughout the protein-coding genome, particularly via tumor whole exome sequencing (WES), has fueled the discovery of many cancer driver genes [1]. However, the vast majority of the genome (~ 98%) is non-coding and contains regulatory elements (e.g. enhancers and promoters) that influence cell/tissue-type specific processes [2]. Methodologically, whole genome sequencing (WGS) allows for genome-wide discovery of somatic variation and may identify novel non-coding driver mutations. Recent studies of non-coding somatic variation identified recurrent mutations in the *TERT* promoter across several cancer types [3, 4] and the *FOXA1* promoter in breast cancer [5]. A variety of computational tools are available for somatic analysis of protein-coding genes [1, 6], while fewer exist for interrogating the non-coding genome. Though studies have devised a variety of analytical strategies for interrogating non-coding somatic mutations, software packages implementing these routines are generally not readily available [4, 5, 7]. Existing tools capable of analyzing this space include OncodriveFML, which permutes variant impact data to assess mutation burden [8], fishHook, which employs a Gamma-Poisson regression framework to model somatic mutation counts along with genomic covariates [9], and MOAT, which permutes regional annotations and mutation locations to identify loci with significant mutation burdens [10].

Here, we describe MutEnricher, a flexible toolset that performs somatic mutation enrichment analysis of both protein-coding and non-coding genomic loci from WGS data. MutEnricher computes both overall mutational burden and “hotspot” enrichments in its analytical routines. MutEnricher is composed of two distinct analysis modules: 1) coding, which identifies genes harboring recurrent non-silent somatic mutations (applicable to both WES and WGS data) and 2) noncoding, which identifies enrichment of somatic variation in user-defined non-coding genomic regions. MutEnricher also implements several methods for computing background mutation rates, including a clustering procedure that groups features (i.e. genes or non-coding regions) by user-defined genomic covariates (e.g. replication timing, sequence GC content, etc.). MutEnricher is implemented in Python (compatible with both major versions 2 and 3), is parallelizable, and is highly configurable to users’ specific datasets.

## Implementation

### Overview

**Fig. 1:**
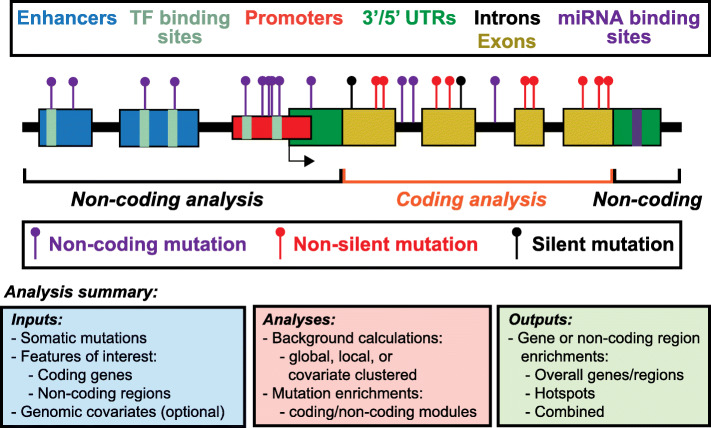
Schematic representation of MutEnricher’s analysis procedures. MutEnricher’s coding module determines enrichment of genic non-silent somatic mutations (red stems) against a background that includes silent (black stems) and non-coding (purple stems) mutations, whereas its noncoding module determines enrichment of non-coding mutations in user-defined genomic intervals, which may include promoters (red region), enhancers (blue regions), etc. The lower boxes summarize MutEnricher’s procedures, describing inputs, analytical steps, and outputs

MutEnricher (Fig. 1) performs somatic mutation enrichment analyses via two distinct modules accessible through the main run script (mutEnricher.py): the coding module, which assesses enrichment of non-silent somatic mutations within coding gene sequences, and the noncoding module, which determines somatic enrichment within user-defined genomic regions (e.g. promoters or enhancers). Both modules compute overall feature (i.e. gene or non-coding region) burden and “hotspot” enrichment significances using binomial statistics by default, with background mutation rates calculated according to one of several user-selected methods. While MutEnricher is designed to work with whole genome somatic mutation calls, the coding module is also capable of analyzing targeted or WES data. Both MutEnricher modules report independent burden and hotspot *p*-values along with combined significance estimates for interrogation by investigators. General run and methodological information are provided here while further details are provided in the Supplementary Information.

### Required inputs and file formats

Somatic mutation data is provided to MutEnricher as a list of bgzip-compressed and tabix-indexed somatic variant call format (VCF) files. Coding gene impact annotations (e.g. via ANNOVAR [11]) are required for the coding module to distinguish non-silent versus silent mutations, while no such annotations are required for the noncoding module. MutEnricher interrogates somatic mutation densities in user-defined features of interest; in the coding module, these are coding genes and are provided to MutEnricher with a gene transfer format (GTF) file, while features of interest in the noncoding module are genomic regions defined in an input BED format file. MutEnricher’s coding module is also capable of accepting mutation annotation format (MAF) files containing mutations derived from targeted or WES data.

### Background mutation rate calculations

MutEnricher implements several methods from which users can select for computing background mutation rates, which are necessary for gene and region enrichment calculations. Three main methods are available: 1) global, 2) local, and 3) covariate clustered. With the global method, gene/region backgrounds are computed as the sum of sample somatic mutation counts within all features divided by the total length; thus, with this method, all features within a sample have the same background rate. For the second method, a local background mutation rate is calculated per-gene/region for each sample. Here, local windows (1–2 Mb in length) are scanned around each feature in each sample and the background mutation rate for the samples’ feature is set to the maximal observed rate from this procedure. The third method clusters features by similarity of user-supplied genomic covariates (e.g. GC content, replication timing, etc.) using affinity propagation [12] and calculates per-sample and per-feature rates from the mutation densities of cluster members. MutEnricher supplies utilities to assist users in creating covariate input files for both modules. Finally, an additional method is implemented that combines the behaviors of the local and covariate clustering methods, whereby features are again grouped by genomic covariates but a wider, local window is scanned when determining mutation densities. For all genes/regions, the final background mutation rate is calculated as the geometric mean of sample-wise rates for all samples possessing at least one foreground somatic mutation (non-silent mutation in coding analysis or any mutation in non-coding analysis) in the features of interest.

### Burden and “hotspot” statistical testing

MutEnricher implements two statistical strategies for determining somatic mutation enrichments. The first method (the default) uses the binomial distribution to determine the significance of observing *n* samples containing somatic mutations in a given feature of length *L* with background probability *p*_*n*_:

$$ {\mathrm{Binomial}}_{\mathrm{p}-\mathrm{value}}={\sum}_{i=n}^N\left(\begin{array}{c}N\\ {}i\end{array}\right){p_n}^i{\left(1-{p}_n\right)}^{\left(N-i\right)} $$

Here, *N* is the total number of samples analyzed and *p*_*n*_ is determined from the estimated nucleotide mutation rate *p* obtained from one of the available background calculation methods and the length of the feature *L*:

$$ {p}_n=1-{\left(1-p\right)}^L $$

In the coding module, *n* is the number of samples with at least one non-silent somatic mutation in a gene, while *n* in the noncoding module is the number of samples with any observed somatic mutation in a region.

MutEnricher also allows users to compute gene/region enrichment statistics using a negative binomial testing strategy that determines the significance of observing *k* mutations within a given feature of length *L* and background mutation rate *p* among *N* samples according to:

$$ {\mathrm{NB}}_{\mathrm{p}-\mathrm{value}}={\sum}_{r=0}^{x-k}\left(\begin{array}{c}k+r-1\\ {}r\end{array}\right){p}^k{\left(1-p\right)}^r $$

In the coding module, *k* is the total number of non-silent somatic mutations found within a gene and *x* is the gene’s coding length multiplied by the total number of tested samples (i.e. *x* = *L* x *N*). In the noncoding module, *k* is the total number of somatic mutations found within the region and *x* is the length of the region multiplied by the total number of samples.

In addition to computing overall gene/region burden enrichments, MutEnricher also finds significant “hotspot” enrichments by progressively grouping somatic mutations within short linear distances (e.g. 50 base pairs, a user-defined parameter) and directly testing these sub-regions for significance (similar to [4]). The noncoding module additionally implements a permutation-based weighted average proximity (WAP) scheme (described in [5]) as another test for mutation clustering. MutEnricher reports both independent burden and hotspot *p*-values along with combined significance estimates using Fisher’s method. All statistical tests are subsequently corrected for multiple hypotheses using the Benjamini-Hochberg FDR procedure [13].

### Datasets, run characteristics, and comparisons to existing tools

We obtained several somatic MAF files from TCGA cohorts and ran MutEnricher’s coding module on these in an exome-specific mode. During these runs, we required candidate hotspots to have at least five somatic mutations from at least three patients. We compared MutEnricher results from each cancer type against results from MutSigCV [14], MutSig2CV [15], fishHook [9], and OncodriveFML [8]. We also obtained breast, liver, and medulloblastoma whole genome somatic mutation data from [16] to test both MutEnricher’s coding and noncoding modules on WGS data. We compared MutEnricher coding results on these data to fishHook and OncodriveFML, and additionally compared noncoding module results to MOAT [10]. Further details are provided in the Supplementary Information and analysis run times on synthetic WGS data are reported in Supplementary Table 1.

## Results and discussion

We ran MutEnricher’s coding module on seven WES-derived mutation datasets from TCGA and compared these results to MutSigCV, MutSig2CV, fishHook, and OncodriveFML significance calls (corrected *p*-values < 0.01). Overall, we observed strong overlap among genes called statistically significant by MutEnricher’s burden testing strategy with those also called by MutSigCV (100% median/~ 76% mean overlap, dataset-wise overlap significance all < 2.8e-4 by hypergeometric test, Supplementary Table 2A). Genes not identified as significant by direct burden testing but significant when hotspots were considered include *KRAS* in BRCA (burden FDR = 1, combined FDR = 4.2e-7) and *BRAF* in GBM (burden FDR = 1, combined FDR = 7.8e-12). We compared MutEnricher’s combined burden and hotspot results to MutSig2CV significance calls, which considers three types of evidence levels, and again found substantial overlap between significant gene calls (69% median/55% mean overlap, Supplementary Table 2B). MutEnricher burden results were also consistent with fishHook results (81.8% median/66.3% mean overlap, Supplementary Table 2C) and, to a lesser degree, with OncodriveFML (62.5% median/53.5% mean overlap, Supplementary Table 2D); these latter results are likely a consequence of the distinct variant impact permutation strategy employed by OncodriveFML. Overall, results from these tools on TCGA lung datasets (LUAD and LUSC) were highly variable (e.g. 539 significant genes in LUAD by MutSigCV, 4 by fishHook); MutEnricher’s consistency with all tools was higher when these cancer types were not considered (Supplementary Tables 2A-D).

**Fig. 2:**
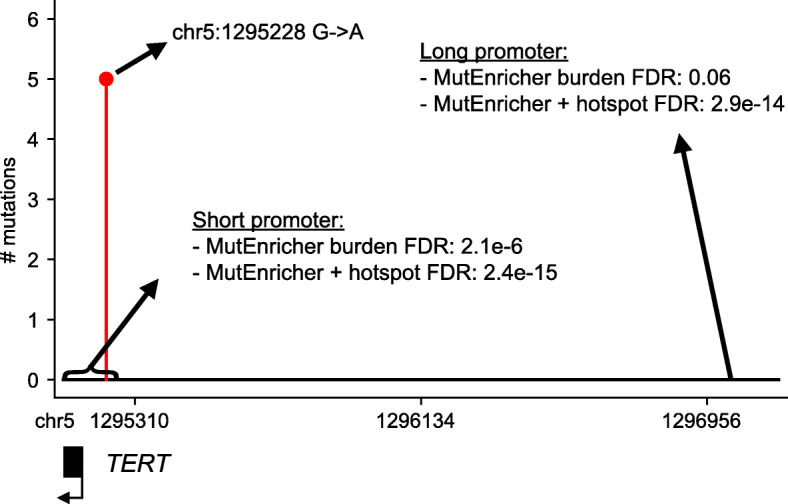
*TERT* promoter region (hg19 chr5:1295105–1,295,262 short region; chr5:1295105–1,297,162 long region) displaying hotspot somatic mutations (chr5:1295228 G➔A) identified in liver cancer whole genome datasets. MutEnricher full region (burden) and full promoter plus hotspot (+ hotspot) significance calls are also displayed

We next tested MutEnricher’s coding and noncoding modules on breast, liver, and medulloblastoma whole genome somatic mutation calls from Alexandrov et al. [16]. We compared coding and non-coding analysis results to fishHook and OncodriveFML and additionally tested non-coding results against MOAT’s annotation-based algorithm. Significantly mutated genes called by MutEnricher and fishHook were highly consistent (87.5% median/84.7% mean overlap), while OncodriveFML results differed from both tools (Supplementary Tables 3A-B). Genes called by MutEnricher and fishHook included *TP53*, *GATA3*, and *PIK3CA* in breast, *TP53* and *ALB* in liver, and *DDX3X* and *SMO* in medulloblastoma. For non-coding analyses, we focused on liver somatic mutations as hepatocellular carcinomas are known to possess recurrent hotspot mutations in the *TERT* promoter [17]. We used two separate definitions for gene promoters in these analyses: 1) 100 basepairs immediately upstream of gene transcription start sites (TSSs) and 2) 2 kilobases upstream of gene TSSs with extension into the 5′ untranslated region (5′ UTR). With the short promoter definition, all tools, with the exception of OncodriveFML, identified the *TERT* promoter as highly statistically significant (Fig. 2, Supplementary Table 4). Five samples in this cohort possess a *TERT* proximal promoter hotspot mutation that creates an ETS binding site [17]. With the longer promoter definition, the reported significance levels for this region dropped for most tools, often beyond thresholds for significance. MutEnricher’s hotspot detection method, however, helped identify this longer *TERT* promoter region as highly significant (combined burden plus hotspot FDR = 2.95e-14). Thus, MutEnricher is less sensitive to specific boundary definitions of non-coding regulatory elements because it interrogates overall regional burdens as well as hotspots.

## Conclusions

Here, we presented MutEnricher, an open source, highly customizable, and well documented toolset that analyzes both coding and non-coding somatic mutation enrichment. We demonstrated general consistency between MutEnricher and other available tools that analyze the coding and/or non-coding genome on a variety of WES and WGS datasets. We also highlighted advantages of MutEnricher’s combined burden and hotspot testing strategies towards driver gene and non-coding element identification. Particularly with respect to non-coding elements, this combined strategy enables MutEnricher to robustly identify mutation recurrence from variable definitions of the same regulatory elements (e.g. “short” versus “long” promoters). In addition, MutEnricher can be run quickly with a variety of parameters, allowing users to test various assumptions and alternative hypotheses. As WGS is increasingly being employed to identify novel somatic alterations, particularly towards the study of many cancers, we believe MutEnricher will be a valuable analytical tool for the research community.

## Availability and requirements

*Project name:* MutEnricher

*Project home page*: https://github.com/asoltis/MutEnricher

*Operating system*: Linux, macOS (source code); Docker-compatible systems

*Programming language*: Python 2 & 3

*License*: MIT license

*Any restrictions to use by non-academics*: None

## Supplementary information

**Additional file 1.** MutEnricher Supplementary Information. MutEnricher supplementary methods and results.

## Data Availability

Synthetic WGS datasets, gene models, and non-coding element definitions are available as example data on the MutEnricher GitHub page (https://github.com/asoltis/mutenricher); TCGA exome MAF files are available from the Broad Institute’s Firehose server (https://gdac.broadinstitute.org/); WGS cancer mutation datasets are available at ftp://ftp.sanger.ac.uk/pub/cancer/AlexandrovEtAl/somatic_mutation_data.

## Abbreviations

WGS: Whole genome sequencing
WES: Whole exome sequencing
VCF: Variant call format
BED: Browser extensible data
GTF: Gene transfer format
MAF: Mutation annotation format
Mb: Megabase
WAP: Weighted average proximity
FDR: False discovery rate
TCGA: The Cancer Genome Atlas
TSS: Transcription start site
UTR: Untranslated region
