# Supplementary material for: MutEnricher: a flexible toolset for somatic mutation enrichment analysis of tumor whole genomes
Source: BMC Bioinformatics. 2020 Jul 31;21:338. doi: 10.1186/s12859-020-03695-z (PMC7393734; doi:10.1186/s12859-020-03695-z)
Supplement: Supplementary file 1 — Additional file 1. MutEnricher Supplementary Information. MutEnricher supplementary methods and results. [file 12859_2020_3695_MOESM1_ESM.pdf]

MutEnricher Supplementary Information

1. MutEnricher Methodological Details

***Features of interest and input mutations**:* In the `coding` module, genes are the features of interest and are defined by protein-coding genes annotated in an input gene transfer format (GTF) file (readily available from various repositories, e.g. UCSC, GENCODE, or ENSEMBL). A gene's total boundary starts from the first position of the first annotated exon and extends to the last position of the last annotated exon for all annotated transcripts of the gene. A gene's *total length* is the length of the total gene boundary; the *coding length* of a gene is the total length of bases residing in annotated coding domain sequences (CDS) within the total gene boundary. Overlapping coding domains (e.g. due to alternatively annotated exons) are merged into a composite set of potential coding domains. In the `noncoding` module, regions are the features of interest and are defined as genomic intervals contained in an input BED-formatted file.

Somatic mutations are provided to MutEnricher with VCF files (or, alternatively, MAF files in some cases). In the `coding` module, annotated mutations are classified as 1) *nonsilent* if they occur in an annotated coding domain and are protein-altering (e.g. missense, frameshift, etc.) or if they alter a splice site (specific nonsilent terms are dependent on the variant annotation method used and user definitions), or 2) *silent* if they occur outside of coding domains or are not protein-altering. In the `noncoding` module, all somatic mutations within each region are considered, i.e. no distinction is made between silent and non-silent events.

***Background mutation rate calculations**:* MutEnricher's statistical models require background somatic mutation rate estimates for every gene/region. Three main methods for gene/regional background mutation rate calculations are available: 1) global, 2) local, and 3) covariate clustered. Methods (1) and (2) are described here and method (3) is described in the next section.

The global background method is the simplest approach. In the `coding` module, background frequencies are computed for each sample as the total number of somatic mutations (silent plus non-silent by default, though this behavior can be adjusted to only consider silent mutations) divided by the *total length* (exons plus introns) of all tested genes. In the `noncoding` module, global per-sample backgrounds are computed as a sample's total number of somatic mutations in all BED-defined intervals divided by the total length of all these regions. Thus, for each gene/region tested, the global per-sample background frequency is constant. This method does not consider local influences or genomic covariates that alter focal mutation frequencies, though may be ideal for cohorts with low overall somatic mutation rates.

In the second method, a local background mutation rate is calculated per-gene/region for each sample. When utilizing this method in the `coding` module, if the total length of a gene is greater than one megabase and the total number of mutations in this region is greater than zero, the local per-sample background is defined as the total number of mutations divided by the gene's total length. Otherwise, somatic VCFs are scanned for mutations in windows of 1-2 Mb around the gene midpoint and background frequencies are computed per window (total mutations in window divided by the window size). The maximum computed background frequency from the various windows defines the local background rate for the gene in that sample. This same local background strategy is used in the `noncoding` module, though no minimum length restriction is applied.

Gene/regional clustering by genomic covariates: The third method for calculating background mutation rates groups features by genomic covariates. Such covariates can include length, sequence content (e.g. GC and CpG frequencies), replication timing, and nearby gene expression. These covariates are provided via an input text file that matches the genes (regions) being considered in the input GTF (BED) file.

Features are clustered by genomic covariates using affinity propagation [1], a clustering algorithm that groups data represented by a graphical model with a "message passing" (or "belief propagation") methodology. Affinity propagation is advantageous in that it is deterministic given the same input data, does not require pre-definition of the total number of expected clusters (as opposed to related methods like k-means or k-medoids), and is comparatively fast when dealing with problems containing large numbers of data points (for example, a user might analyze tens of thousands of genes/regions). The input to affinity propagation is a graph with edges scored by the "similarity" between nodes. MutEnricher calculates similarity as the weighted negative squared distance between feature covariates:

$$s = -\sum_{i}^{n} w_i (p_i - q_i)^2$$

where p and q are two feature vectors (e.g. for a specific gene) with n associated genomic covariates and w_i is the weight of covariate i in the similarity calculation (normalized such that their sum is equal to one). If all weights are equal, this reduces to the average squared distance; otherwise, weights influence the relative importance of each covariate in the similarity calculations. This weighting scheme can be used to adjust the relative importance of specific features or normalize the influence of covariates describing similar information. For example, users may provide information from multiple datasets describing gene expression levels; weights can be used to prevent over emphasis of gene expression in the clustering by down weighting the individual datasets. Prior to similarity calculations, each covariate is mean-centered and variance-scaled (i.e. z-scored) across the feature dimension. Affinity propagation does not require that $s_{i\text{->}j} = s_{j\text{->}i}$; however, these values are indeed equal in our application.

While affinity propagation does not *a priori* require a target number of final clusters, the resulting number of clusters is influenced by a "self-similarity" parameter. MutEnricher sets the self-similarity to the median of all calculated similarities. In cases where the algorithm does not converge, the self-similarity parameter is slightly perturbed and re-run with the same similarities until convergence is achieved (users can also alter the convergence criteria of affinity propagation runs). Each feature's per-sample background rate is then calculated as the total number of mutations contained in all the feature regions that are members of its cluster (including itself) divided by the total length of all features within the cluster. In cases where a feature is a singleton or belongs to a very small cluster (e.g. < 3 cluster members, a user adjustable parameter), MutEnricher uses method (2) above to calculate a local background frequency for the feature of interest.

Combined covariate clustering plus local background mutation rate method: MutEnricher also implements an additional "fourth" background mutation rate calculation method, which combines the local and covariate clustering methods (i.e. methods 2 & 3) described above. With this method, gene/region covariate clusters are identified with affinity propagation and background mutation rates are calculated with respect to these clusters as described; the difference here is that sample-wise feature

rates are computed using the local strategy, whereby the mutation density in a 1-2 Mb window around each feature is used to estimate the background. This method can be appropriate in cases where small length features are of interest, which may produce overly strict background rate estimates leading to overly conservative p-values when the default covariate clustering strategy is implemented.

Statistical testing: MutEnricher implements two statistical testing strategies from which users can choose to determine somatic mutation enrichment p-values:

Binomial testing strategy: MutEnricher's binomial testing strategy (the default method) determines the significance of observing n samples among N total containing somatic mutations in a given feature of length L with background probability p_n according to:

$$\text{Binomial}_{\text{p-value}} = \sum_{i=n}^{N} \binom{N}{i} p_n{}^i (1-p_n)^{(N-i)}$$

Here, p_n is determined from the estimated nucleotide mutation rate obtained from one of the available background calculation methods (p) and the length of the feature L:

$$p_n = 1 - (1-p)^L$$

In the `coding` module, n is the number of samples containing at least one non-silent somatic mutation in a gene and L is equal to the gene's coding length. In the `noncoding` module, n is the total number of samples containing at least one somatic mutation of any type within the region and L is the length of the region. In both modules, the nucleotide background mutation probability p is the geometric mean of the per-sample background mutation rates for all samples with at least one foreground mutation in the gene/region. MutEnricher uses the `binom_test` function in the Python SciPy package to obtain binomial test p-values according to: `binom_test(n, N, pn, alternative='greater')`.

Negative binomial testing strategy: MutEnricher's negative binomial testing strategy determines the significance of observing k mutations within a given feature of length L and background mutation rate p among N samples according to:

$$\text{NB}_{\text{p-value}} = \sum_{r=0}^{x-k} \binom{k+r-1}{r} p^k (1-p)^r = I_p(k, x-k+1)$$

where I_p is the regularized incomplete beta function:

$$I_p(a,b) = \frac{\text{B}(p;a,b)}{\text{B}(a,b)} = \frac{\int_0^p t^{a-1}(1-t)^{b-1}dt}{\int_0^1 t^{a-1}(1-t)^{b-1}dt}$$

In the `coding` module, k is the total number of non-silent somatic mutations found within a gene and x is the gene's coding length multiplied by the total number of tested samples (i.e. $x = L$ x N). In the `noncoding` module, k is the total number of somatic mutations found within the region and x is the length of the region multiplied by the total number of samples. In both modules, the background mutation probability p is the geometric mean of the per-sample background mutation rates for all samples with at least one foreground mutation in the gene/region. MutEnricher uses the `betainc` function in the Python SciPy package, which calculates the regularized incomplete beta function with

parameters a, b, and p, to obtain negative binomial test p-values.

"Hotspot" detection methods and multiple hypothesis corrections: In addition to overall feature enrichments, MutEnricher incorporate methods to test for significant local, or "hotspot," mutation densities. In both the `coding` and `noncoding` modules, MutEnricher performs a hotspot enrichment procedure that progressively aggregates mutations in close proximity (e.g. 50 base pairs) and tests for enrichment significance of the n_{hs} samples with such mutations using a binomial test or the significance of k_{hs} variants with the negative binomial model described above (similar to the method employed in [2]). In this case, L is equal to the candidate hotspot length and the nucleotide background mutation rate p is calculated from the same features used in the burden rate calculations, though only samples containing a mutation within the candidate hotspot window are considered. Candidate hotspots are considered for testing if they contain at least X somatic mutations from Y samples (e.g. at least 3 mutations from 3 samples, adjustable by the user to allow for more permissive or more conservative definitions; we generally recommend X should be at least > 1 and Y = 3 for cohort sizes of ~100 samples). All candidate hotspots meeting these criteria, along with their associated significance results, are then reported. In coding analyses, only non-silent mutations are considered and candidate hotspots are discovered per exon (i.e. hotspots do not span multiple exons), while all mutations are considered in non-coding analyses.

In the `noncoding` module, we additionally employ a weighted average proximity (or WAP) method to test for significant clustering of mutations (as employed in [3], based on [4]). The test statistic for this procedure is:

$$\mathrm{WAP} = \sum_{i \neq j} n_i n_j e^{\frac{-d_{ij}^2}{2\tau^2}}$$

where d is the distance (in genomic coordinates) between positions i and j harboring somatic mutations, n_i and n_j are the mutation counts at the two positions, and τ is the exponential distance constant (e.g. typically set to 6). The WAP statistic is summed over all pairs of positions i and j except when $i = j$.

A permutation procedure is used to evaluate the statistical significance of the WAP test statistic. In this procedure, permuted test statistics (e.g. WAP_p) are computed with the above function after randomization of mutation positions within the region, though retaining the total number of mutations at each position. To speed computation of permuted p-values, we employ the stopping criteria described in [5] that provides a principled test for terminating permutation computations once a desired level of accuracy is achieved:

$$2 * 1.96 * \sqrt{\frac{N - n + 1}{(N + 3)(n + 1)}} < \theta$$

Here, n is the number of permuted test statistics that are greater than or equal to the true WAP value, N is the current number of permutations, and θ describes the desired accuracy of the estimated p-value based on its confidence interval. We set $\theta = 0.25$ by default. For each region, we terminate computations if a) the above relationship is satisfied after running a minimum of 1000 permutations or b) 1e6 permutations are run.

For both modules, MutEnricher reports both overall feature (gene/region) burden as well as combined burden plus hotspot significance levels. Combined p-values are calculated with Fisher's method. In the `noncoding` module, the burden p-value is reported as the combined overall regional and WAP significance levels by default. Users can alter this behavior by setting the `--no-wap` flag to disable the WAP procedure, reporting only the overall regional significance as the burden p-value. Both modules independently report candidate hotspots and associated significance levels in separate output tables. The Benjamini-Hochberg FDR procedure [6] is used to correct for multiple hypotheses in all output tables, considering all gene/regions with at least one foreground mutation (i.e. non-silent mutations in genes, any mutation in non-coding regions) in burden/combined output tables or all candidate hotspots in hotspot outputs.

Using whole exome sequencing data and MAF input: MutEnricher is designed for whole genome sequencing datasets, though analysis of targeted or exome sequencing data is also possible with the `coding` module. This data type can be provided with VCF inputs or with Mutation Annotation Format (MAF) files, the latter via the `--maf` option. When analyzing WES data, users should set the `--exome-only` flag, which instructs the program to only consider the exome spaces of genes in its computations. When analyzing other targeted sequencing data, users can define the covered genomic space with the mappability options (e.g. `-m`). With MAF input, only the global and covariate clustered background methods are usable, as the local method requires indexed VCFs to scan regions surrounding the gene of interest for mutations. In addition, users can adjust the background mutation model to only consider "silent" mutations with the `--bg-vars-type` flag; the default behavior is to consider all mutations within the gene's total space, which may produce overly conservative p-values when not considering intronic regions.

Additional features and run options: MutEnricher provides options that users can specify to best match their research goals. The `coding` module requires that somatic VCF mutations are annotated such that non-silent mutations can be identified. Some somatic variant callers provide such annotations (e.g. Illumina's HiSeq Analysis Software); alternatively, third-party software tools (e.g. ANNOVAR [7]) can be used for annotation. To account for formatting differences between annotation engines, users can explicitly define which VCF fields and terms qualify as non-silent variants by providing a simple text file with the `--anno-type` option. This option can also be set to one of several pre-defined annotation terms sets, for example ANNOVAR refGene annotations.

Both analysis modules implement covariate clustering methods for background mutation rate calculations. With large feature spaces (e.g. >20,000), this procedure can add considerable run time to the analysis (see **Supplementary Table 1**). As mitigation, users can perform this clustering once and re-use the output in subsequent runs on the same or independent data. In the `coding` module specifically, users can select the `--by-contig` option to break up the clustering problem by contigs (e.g. chromosomes). This procedure dramatically reduces the problem scale and run time (this option is implemented by default in the `noncoding` module), though the calculations differ from the full analysis as background rates are computed with respect to features on each chromosome. The `coding` module also allows users to reduce the total number of analyzed genes with the `-g` option (applicable, for example, in cases where users wish to only consider expressed genes in their samples). Lastly, MutEnricher allows users to provide their own custom pre-computed gene or region clusters.

A common issue with any whole genome sequencing experiment is the reliability of genotype/variant calling in poorly mappable regions (due to repeats, CpG islands, etc.). It is advisable to carefully consider which variants are included in any analyses. "Blacklist" variants can be supplied to

MutEnricher as a text file; however, it is recommended to pre-filter input mutation files (e.g. with bcftools) if these lists become large in order to avoid large RAM usage, especially when using parallelization. As a complementary or additional option, users can supply a list of mappable genomic regions with an indexed BED file. With such input, MutEnricher will only analyze variants within intervals overlapping these mappable regions and adjust gene/region lengths accordingly during significance testing. MutEnricher also includes options to define the "hotspot" distance threshold and the number of processors for parallel runs.

External Python modules used by MutEnricher: MutEnricher uses the following non-standard Python libraries:

- NumPy (www.numpy.org)
- SciPy (https://scipy.org)
- Cython (cython.org)
- pysam (pysam.readthedocs.io/en/latest/index.html)
- cyvcf2 (https://github.com/brentp/cyvcf2) [8]

2. Additional Supplementary Methods

Annotation files and MutEnricher run details: We obtained human hg19 RefSeq gene model annotations from the UCSC Table Browser tool [9] (refFlat GTF, downloaded on August 29, 2017). For "long" gene promoter annotations, we used a window of two kilobases upstream to the end of the annotated 5' UTR for all coding gene transcripts from the refFlat GTF and used BEDTools [10] to merge overlapping intervals. For "short" gene promoter annotations, we obtained hg19 coordinates 100 bp upstream of annotated GENCODE coding transcripts from http://files.gersteinlab.org/public-docs/2017/08.20/coding.tss.bed.

For all runs of the MutEnricher `coding` module in this study, we used the covariate clustering method for background mutation rate calculations. For MutEnricher `noncoding` runs, we used the combined covariate clustering and local background method. For each feature, we used total region length, coding region length (coding analysis only), GC content, CpG content, and replication timing as covariates. For replication timing information, we obtained human Repli-seq data from the ENCODE project page (https://www.encodeproject.org/). We converted the downloaded bigWig files to bedGraph format and, for each analyzed feature, obtained a replication time value as either 1) the average value from all overlapping windows with the feature of interest or, 2) in cases where no overlapping Repli-seq interval was found, progressively expanded the search window until at least one interval was observed and used this value (or the mean if multiple overlaps were identified). If no overlapping interval was found after a maximum search window was used, a NaN value was reported. MutEnricher's clustering module handles missing data by imputing with the mean value for the specific covariate.

Synthetic WGS datasets: We created 100 synthetic somatic VCF files by inserting random mutations throughout the genome at a rate of two mutations per megabase. We also introduced three true positive test cases, two coding and one non-coding, by inserting known "non-silent mutations" within the *KRAS* and *TP53* genes (target cohort frequencies of 30% and 90%, respectively) along with random mutations throughout the *TERT* promoter region (one kilobases upstream to 5' UTR end, 40% target frequency). We annotated these variants with ANNOVAR [7] using hg19 RefGene models.

TCGA exome datasets: We obtained published TCGA somatic MAF files along with associated MutSigCV and MutSig2CV results from the Broad Institute's Firehose database

(https://gdac.broadinstitute.org/) for several cancer types: breast invasive carcinoma (BRCA), glioblastoma multiforme (GBM), glioma (GBMLGG), lung adenocarcinoma (LUAD), lung squamous cell carcinoma (LUSC), ovarian serous cystadenocarcinoma (OV), and prostate adenocarcinoma (PRAD). For each dataset, we converted gene symbols to current HUGO Gene Nomenclature Committee (HGNC) symbols using the multi-symbol checker tool available at www.genenames.org [11]. For MutEnricher runs on this data, we set the `--exome-only` flag to true and required at least 5 mutations from at least 3 patients for identification of candidate hotspots.

Whole genome datasets: We obtained whole genome somatic mutation data for breast, liver, and medulloblastoma cancers from ftp://ftp.sanger.ac.uk/pub/cancer/AlexandrovEtAl/somatic_mutation_data [12]. We annotated variants with ANNOVAR hg19 refGene models and formatted somatic mutation input files for appropriate use with each of the tested analysis tools.

Run details for additional tools:

- *MutSigCV and MutSig2CV*: We obtained available analytical results the Broad Institute's Firehose database for these tools as described above.

- *fishHook*: We ran fishHook (version 0.1) [13] within version 3.5.0 of R. For all analyses, mutations were either read from MAF files or text files containing WGS mutations and variant impact information. For gene and CDS information, we used GENCODE version 19 data supplied with the fishHook package. Prior to coding analyses, we subset genes to those annotated as "`protein_coding`", padded CDS definitions -/+ 10 bp to allow for potential splice site mutations, and subset mutations to non-silent; these values served as the hypotheses, eligible territory, and events in the fishHook model, respectively. For non-coding analyses, hypotheses were set to promoter regions (see above for definitions), events were set to all somatic mutations, and eligible territory was restricted to the promoter sequences. For both coding and non-coding analyses, keratinocyte replication timing data and sequence GC content provided with the software package were used as covariates when running the fishHook model.

- *OncodriveFML*: We ran OncodriveFML (version 2.3.0) [14] with CADD (version 1.3) [15] variant impact scores. For elements, we used hg19 refGene CDS regions for coding analyses and gene promoters for non-coding analyses. All other run parameters were set to default values, which included consideration of indels using the 'max' simulation method for scoring.

- *MOAT*: We ran MOAT's (version 1.0) [16] annotation-based algorithm with the CPU command line script `moat_a_cpu`, with hg19 gene promoter definitions as the input annotations, 10,000 permutations, *dmin* = 2000, *dmax* = 100000, and the hg19 genome blacklist file provided with the software package.

Disclaimer: The contents of this publication are the sole responsibility of the author(s) and do not necessarily reflect the views, opinions or policies of Uniformed Services University of the Health Sciences (USUHS), the Department of Defense (DoD), The Henry M. Jackson Foundation for the Advancement of Military Medicine, Inc., or the U.S. Government.

Supplementary Tables

Module	BMR type	Clustering type	Clustering run time (min.) (single/multithread)	Total run time (min.) (single/multithread)
Coding	Global			1.59 / 0.55
Coding	Local			2.33 / 0.71
Coding	Clustering	Precompute	0.01 / 0.01	1.88 / 0.84
Coding	Clustering	By contig	3.18 / 1.05	4.68 / 1.68
Coding	Clustering	All genes	96.78 / 96.88	98.43 / 97.70
Noncoding	Global			0.91 / 0.43
Noncoding	Local			1.46 / 0.50
Noncoding	Clustering	Precompute	0.13 / 0.06	1.03 / 0.50
Noncoding	Clustering	By contig	3.76 / 1.08	4.67 / 1.51

Supplementary Table 1. MutEnricher run times on synthetic somatic data. MutEnricher run times on 100 synthetic somatic datasets for various analysis types at different settings. “Module” indicates whether or not the `coding` or `noncoding` module was run; “BMR type” indicates the background mutation rate methodology used (global, local, or covariate clustered); “Clustering type” indicates, if the covariate clustering background method was used (gray cell if not used), whether or not precomputed clusters were used (precompute), clustering was performed by contig (e.g. by chromosome), or all features were used (all genes, coding analysis only); “Clustering run time” indicates, if covariate clusters were used, the run times (in minutes) for this portion of the analysis; “Total run time” indicates the total analysis run times (in minutes). Single versus multithread indicates run times from analyses using either a single processor or 10 processors (multithread). Covariate clustering with affinity propagation was performed on 19,332 genes for coding analyses and 21,324 promoter regions for non-coding analyses. Note clustering time speed-ups are observed when performing analysis by contig as this takes advantage of parallelization effectively. The clustering procedure is the major rate limiting step in coding analyses run with all genes. However, these results can be used in subsequent runs on the same or independent data with the precompute option.

TCGA dataset	# MutEnricher significant genes (Burden FDR < 0.01)	# MutSigCV significant genes (Burden FDR < 0.01)	Overlap (%)	Overlap significance
BRCA	8	25	100	3.55e-24
GBM	9	10	77.8	3.23e-23
GBMLGG	11	28	100	1.15e-32
LUAD	322	539	35.1	8.68e-92
LUSC	28	12	21.4	6.75e-15
OV	1	5	100	2.74e-4
PRAD	3	6	100	1.97e-11
**Mean (w/out lung)**			76.3 (95.6)	
**Median (w/out lung)**			100 (100)	

Supplementary Table 2A. MutEnricher overall gene burden results on TCGA datasets compared to MutSigCV results. MutEnricher was run on MAF files obtained from the Broad Institute's Firehose database for the above cancer datasets. Overlap is defined as the number of genes called significant by MutEnricher (FDR < 0.01, column 2) that are also called significant by MutSigCV (FDR < 0.01, column 3) over the total number of genes called significant by MutEnricher. Overlap significance was calculated using the right-tail hypergeometric test assuming a background of 18,251 genes. Mean and median values in parentheses represent calculations excluding overlaps with lung datasets (LUAD and LUSC). Sample counts: BRCA: 988; GBM: 283; GBMLGG: 799; LUAD: 533; LUSC: 177; OV: 466; PRAD: 498.

TCGA dataset	# MutEnricher significant genes (Combined FDR < 0.01)	# MutSig2CV significant genes (Combined FDR < 0.01)	Overlap (%)	Overlap significance
BRCA	19	31	73.7	5.88e-37
GBM	11	16	81.8	1.01e-27
GBMLGG	29	56	69.0	1.12e-45
LUAD	645	25	2.9	2.91e-23
LUSC	41	10	17.1	2.0e-17
OV	2	5	50.0	5.48e-4
PRAD	8	19	87.5	3.0e-21
**Mean (w/out lung)**			54.6 (72.4)	
**Median (w/out lung)**			69.0 (73.7)	

Supplementary Table 2B. MutEnricher combined burden and hotspot significance results on TCGA datasets compared to MutSig2CV results. MutEnricher was run on MAF files obtained from the Broad Institute's Firehose database for the above cancer datasets. Overlap is defined as the number of genes called significant by MutEnricher (FDR < 0.01, column 2) that are also called significant by MutSig2CV (FDR < 0.01, column 3) over the total number of genes called significant by MutEnricher. Overlap significance was calculated using the right-tail hypergeometric test assuming a background of 18,251 genes. Mean and median values in parentheses represent calculations excluding overlaps with lung datasets (LUAD and LUSC). Sample counts: BRCA: 988; GBM: 283; GBMLGG: 799; LUAD: 533; LUSC: 177; OV: 466; PRAD: 498.

TCGA dataset	# MutEnricher significant genes (Burden FDR < 0.01)	# fishHook significant genes (Burden FDR < 0.01)	Overlap (%)	Overlap significance
BRCA	8	12	100	8.47e-28
GBM	9	7	66.7	7.04e-21
GBMLGG	11	9	81.8	4.28e-32
LUAD	322	4	1.2	6.87e-8
LUSC	28	7	12.9	1.12e-10
OV	1	1	100	5.05e-5
PRAD	3	4	100	3.09e-12
**Mean (w/out lung)**			66.3 (89.7)	
**Median (w/out lung)**			81.8 (100)	

Supplementary Table 2C. MutEnricher overall gene burden results on TCGA datasets compared to fishHook results. MutEnricher was run on MAF files obtained from the Broad Institute's Firehose database for the above cancer datasets. Overlap is defined as the number of genes called significant by MutEnricher (FDR < 0.01, column 2) that are also called significant by fishHook (FDR < 0.01, column 3) over the total number of genes called significant by MutEnricher. Overlap significance was calculated using the right-tail hypergeometric test assuming a background of 19,796 genes. Mean and median values in parentheses represent calculations excluding overlaps with lung datasets (LUAD and LUSC). Sample counts: BRCA: 988; GBM: 283; GBMLGG: 799; LUAD: 533; LUSC: 177; OV: 466; PRAD: 498.

TCGA dataset	# MutEnricher significant genes (Burden FDR < 0.01)	# OncodriveFML significant genes (Burden FDR < 0.01)	Overlap (%)	Overlap significance
BRCA	8	9	62.5	1.84e-14
GBM	9	5	55.6	3.29e-16
GBMLGG	11	13	72.7	2.96e-22
LUAD	322	14	2.5	9.04e-9
LUSC	28	6	14.3	1.37e-9
OV	1	4	100	4.67e-4
PRAD	3	3	66.7	2.45e-7
**Mean (w/out lung)**			53.5 (71.5)	
**Median (w/out lung)**			62.5 (66.7)	

Supplementary Table 2D. MutEnricher overall gene burden results on TCGA datasets compared to OncodriveFML results. MutEnricher was run on MAF files obtained from the Broad Institute's Firehose database for the above cancer datasets. Overlap is defined as the number of genes called significant by MutEnricher (FDR < 0.01, column 2) that are also called significant by OncodriveFML (FDR < 0.01, column 3) over the total number of genes called significant by MutEnricher. Overlap significance was calculated using the right-tail hypergeometric test assuming a background of 8,564 genes. Mean and median values in parentheses represent calculations excluding overlaps with lung datasets (LUAD and LUSC). Sample counts: BRCA: 988; GBM: 283; GBMLGG: 799; LUAD: 533; LUSC: 177; OV: 466; PRAD: 498.

**Alexandrov *et al.* dataset**	**# MutEnricher significant genes (Burden FDR < 0.01)**	**# fishHook significant genes (Burden FDR < 0.01)**	**Overlap (%)**	**Overlap significance**
Breast	8	7	87.5	3.39e-26
Liver	2	2	100	5.1e-9
Medulloblastoma	3	2	66.7	1.53e-8
**Mean**			84.7	
**Median**			87.5	

Supplementary Table 3A. MutEnricher overall gene burden results on WGS datasets compared to fishHook results. MutEnricher was run on WGS cancer datasets from Alexandrov *et al.* [12]. Overlap is defined as the number of genes called significant by MutEnricher (FDR < 0.01, column 2) that are also called significant by fishHook (FDR < 0.01, column 3) over the total number of genes called significant by MutEnricher. Overlap significance was calculated using the right-tail hypergeometric test assuming a background of 19,796 genes. Sample counts: Breast: 119; Liver: 88; Medulloblastoma: 100.

**Alexandrov *et al.* dataset**	**# MutEnricher significant genes (Burden FDR < 0.1)**	**# OncodriveFML significant genes (Burden FDR < 0.1)**	**Overlap (%)**	**Overlap significance**
Breast	9	0	N/A	N/A
Liver	2	0	N/A	N/A
Medulloblastoma	8	11	62.5	1.39e-8
**Mean**			62.5	
**Median**			62.5	

Supplementary Table 3B. MutEnricher overall gene burden results on WGS datasets compared to OncodriveFML results. MutEnricher was run on WGS cancer datasets from Alexandrov *et al.* [12]. Overlap is defined as the number of genes called significant by MutEnricher (FDR < 0.1, column 2) that are also called significant by OncodriveFML (FDR < 0.1, column 3) over the total number of genes called significant by MutEnricher. Overlap significance was calculated using the right-tail hypergeometric test assuming a background of 740 genes. Sample counts: Breast: 119; Liver: 88; Medulloblastoma: 100.

**Analysis tool**	**Significance metric**	**Significance value – short promoter**	**Significance value – long promoter**
MutEnricher burden	FDR	2.14e-6	0.064
MutEnricher burden + hotspot	FDR	2.35e-15	2.95e-14
fishHook	FDR	2.9e-5	0.11
OncodriveFML	FDR	0.82	0.99
MOAT-a	Permutation p-value	0	0

Supplementary Table 4. MutEnricher's and other tools' *TERT* promoter results on liver cancer whole genome samples. Liver cancer data *TERT* promoter region (chr5:1295105-1295262 short region; chr5:1295105-1297162 long region) significance calls from MutEnricher (burden alone and burden + hotspot analyses), fishHook, OncodriveFML, and MOAT-a. Abbreviations: FDR – false discovery rate.

REFERENCES

1. Frey, B.J. and D. Dueck, *Clustering by passing messages between data points.* Science, 2007. **315**(5814): p. 972-6.
2. Weinhold, N., et al., *Genome-wide analysis of noncoding regulatory mutations in cancer.* Nat Genet, 2014. **46**(11): p. 1160-5.
3. Rheinbay, E., et al., *Recurrent and functional regulatory mutations in breast cancer.* Nature, 2017. **547**(7661): p. 55-60.
4. Kamburov, A., et al., *Comprehensive assessment of cancer missense mutation clustering in protein structures.* Proc Natl Acad Sci U S A, 2015. **112**(40): p. E5486-95.
5. Getz, G., Gould, J., Monti, S., *Boosting permutation tests for marker selection*. 2006, Broad Institute Publications.
6. Benjamini, Y., Hochberg, Y., *Controlling the false discovery rate: a practical and powerful approach to multiple testing.* Journal of the Royal Statistical Society. Series B, 1995. **57**(1): p. 289-300.
7. Wang, K., M. Li, and H. Hakonarson, *ANNOVAR: functional annotation of genetic variants from high-throughput sequencing data.* Nucleic Acids Res, 2010. **38**(16): p. e164.
8. Pedersen, B.S. and A.R. Quinlan, *cyvcf2: fast, flexible variant analysis with Python.* Bioinformatics, 2017. **33**(12): p. 1867-1869.
9. Karolchik, D., et al., *The UCSC Table Browser data retrieval tool.* Nucleic Acids Res, 2004. **32**(Database issue): p. D493-6.
10. Quinlan, A.R. and I.M. Hall, *BEDTools: a flexible suite of utilities for comparing genomic features.* Bioinformatics, 2010. **26**(6): p. 841-2.
11. Gray, K.A., et al., *Genenames.org: the HGNC resources in 2015.* Nucleic Acids Res, 2015. **43**(Database issue): p. D1079-85.
12. Alexandrov, L.B., et al., *Signatures of mutational processes in human cancer.* Nature, 2013. **500**(7463): p. 415-21.
13. Imielinski, M., G. Guo, and M. Meyerson, *Insertions and Deletions Target Lineage-Defining Genes in Human Cancers.* Cell, 2017. **168**(3): p. 460-472 e14.
14. Mularoni, L., et al., *OncodriveFML: a general framework to identify coding and non-coding regions with cancer driver mutations.* Genome Biol, 2016. **17**(1): p. 128.
15. Rentzsch, P., et al., *CADD: predicting the deleteriousness of variants throughout the human genome.* Nucleic Acids Res, 2019. **47**(D1): p. D886-D894.
16. Lochovsky, L., J. Zhang, and M. Gerstein, *MOAT: Efficient Detection of Highly Mutated Regions with the Mutations Overburdening Annotations Tool.* Bioinformatics, 2017.
